# Epizelus (Hdt 6.117): A Medical History Critique and Reappraisal

**DOI:** 10.1093/shm/hkaf006

**Published:** 2025-02-17

**Authors:** James C Ford

**Affiliations:** University of Liverpool, Liverpool, UK

**Keywords:** Herodotus, Greek religion, Marathon, combat trauma, medical history

## Abstract

Most commentators have argued that the ‘curious’ tale of Epizelus is the first historical example of anxiety-related combat trauma. While the capacity to experience combat-related trauma is universal across human societies, focussing on whether Epizelus can be diagnosed with a specific contemporary traumatic condition is presentist and misguided. Instead of engaging in disputes about diagnosis, historians are better placed providing a close reading and dissection of the source material informed by historical and cultural historiography, raising new questions and offering a synthesis and analysis that can then be used responsibly by medical scholars. By doing so, the Epizelus episode can be recontextualised as an epiphany of a god: a story with deep meaning for the Athenians by the time that Herodotus was writing in the late fifth-century BC.

In the battle of Marathon some 6400 of the Persians were killed, while the Athenians lost 192. That was how many fell on either side. [2] It happened in that place that the following marvellous thing occurred: an Athenian soldier, Epizelus the son of Cuphagoras, was fighting bravely in the melee when suddenly he was deprived of both his eyes, neither having been hit in the body nor with a missile, and from that time he continued blind as long as he lived. [3] In speaking about what happened to him, I heard that he used to say something like this: that he thought he was opposed by a man of great stature in heavy armour, whose beard overshadowed his shield; but the phantom slipped by, and killed the man at his side. These things I learned Epizelus was saying.^1^

The short story of Epizelus has been described as the first recorded instance of any number of conditions arising from an adverse psychological reaction to combat, such as ‘shell shock’,^2^ ‘war neurosis’,^3^ ‘battle hysteria’ or ‘hysterical blindness’,^4^ ‘battle fatigue’,^5^ ‘chronic mental symptoms’,^6^ and ‘conversion blindness’ or ‘conversion disorder’,^7^ in which otherwise unexplained neurological symptoms are traced back to a psychological trigger. It has most commonly been described as post-traumatic stress disorder (PTSD), a complex psychiatric injury with associated long-term anxiety disorder developed after (a) traumatic event(s) in which the individual is subject to significant culturally specific physical, cognitive, emotional, social or spiritual stressors.^8^ Epizelus holds considerable interest in medical circles, with a range and diversity of references to the passage in this context going back over 100 years. Since the First World War, this short passage in Herodotus’ *Histories* 6.117 has been primarily valued for its place in medical history.

In this article, I argue that historical focus has been lost or subsumed in discussion of this passage, as historians have been caught up in disputes rooted in diagnosis by medical professionals. I aim to give a close analysis of the primary source material and historiography in history that is accessible to scholars in history and health. This is an attempt to move the discussion in history beyond diagnosis, to raise different and overlooked aspects of the episode, and to empower scholars studying the history of traumatic responses in evaluating the significance and broader meaning of the story.

Despite much interest and more than a century of scholarship, there is no consensus either on a specific diagnosis for Epizelus or if one is possible or valid.^9^ There are fundamental, unavoidable problems with using ancient texts like this for individual diagnosis. Even if Herodotus did record suitable information, he recorded it in an unsystematic way, and the ancient author’s methodology—from his distinctive blending of myth and history to his controversial and unreliable use of sources—prevents his text from being used as a source of reliable factual information, which in turn impedes the use of his work in clinical diagnosis, as Griffiths has cautioned in the case of the Spartan King Cleomenes:

There is no clear watershed to be found between the Pharaoh Pheros, who cured his blindness with the aid of a chaste woman’s urine, and the historical King Kleomenes, nowhere that we can stand and say ‘folktale this side, history that’. In our present state of knowledge, I should certainly regard any attempt to diagnose the particular clinical variety of the alleged psychosis of King Kleomenes as premature.^10^

This should not be used to infer that combat trauma did not exist in the ancient world: ancient combatants did experience traumatic responses to battlefield experiences, even if we cannot easily access them in a quantifiable diagnostic framework.^11^ Jason Crowley has argued that respective conditions mean that the Athenian hoplite cannot have suffered from PTSD; however, as Alan Greaves observed, the conditions for the creation of trauma are far broader than specific modern military conditions, and diagnoses of PTSD can now include a variety of civilian experiences such as car accidents, rape and sexual assault, and natural disaster.^12^ While certain specific psychogenic conditions such as PTSD have been argued to be highly contextual, and their universality remains a hotly contested issue, clinicians have increasingly recognised a much broader pool of trauma-related conditions could have occurred across history and culture, both on and off the battlefield.

The capacity to experience trauma, including from combat, is universal across human societies, and the conditions in its creation could have been met in ancient combat.^13^ It has persuasively been argued that combat trauma was likely far more common in ancient societies, which required routine military participation of adult males in close-quarters combat, and as such it was conceived of within communal frameworks and culturally available terms of heroism rather than pathologised in individuals.^14^ Consequently, combat trauma can be traced on a societal scale through echoes in literature and culture.^15^ This can help us access the ‘hidden voices’ of the traumatised that are often lost in diagnostic analyses, both as individuals and communities, and understand the meaning and significance of trauma in the ancient world.^16^ Starting with a sensitive historical method, rather than diagnostic applied science, is crucial to understanding the nature of trauma in the ancient world and deploying this kind of source material in an ethical and valid way.

Approaches to the episode have been bound up in ‘Whig history’: a common approach to history in health sectors, in which constant progress over time is assumed; as well as precursor history (or Great Man Theory), with its focus on key individuals; and the mining of complex source material for simple lessons or analogues.^17^ Historians have been drawn into debates built on these faulty assumptions and often expected to give simple answers restricted to the medical or scientific scope.^18^ Instead, historians operating in health fields can more effectively work to highlight fundamental assumptions and raise deeper and broader questions, to complicate assumedly ‘unproblematic’ issues, change the nature of the discussion, help to understand difference and change and offer an effective view of the bigger picture.^19^ Particularly useful is the contextualisation of source material in culture, society, and power dynamics: after all, the health of populations is the product of war, religion, economics and migration, so an adequate historical treatment is invaluable for understanding the social determinants of disease.^20^ Historical methods offer distinct advantages, including the synthesis of robust historiography often missing from health discussions and the capture of new aspects of the material or new issues for consideration.^21^ Historians are not only adept at critiquing the use of complex raw material in quantitative research but also at helping to process it into usable quantitative data.^22^

To offer these advantages, analysis must rest on robust historical research methods: methodical source investigation and synthesis rather than hypothesis validation.^23^ In this case, scholars have constructed a debate largely concerned with hypothesis validation of the view that Epizelus had PTSD or some other adverse psychological reaction to combat and historians have often slipped into verification or falsification of this theory rather than insisting on being a critical third party; the ‘marginal natives’; ‘living among the tribe’; a ‘stranger and a friend’ to medical discussions of the story.^24^

## Contextualising the Passage

The story of Epizelus was a common local legend in Athens by the end of the fifth-century BC, and it was well-known long before it was written down by the historian Herodotus in his *Histories* (usually dated to 430–424 BC, with some argument for 414 BC, or even after 404BC),^25^ whose account serves as our main source of information on the episode.^26^ The tale is also recorded by the Plutarchian writer of some minor *Lives* of *Datis and Hasdrubal*, which is the fullest surviving account other than Herodotus’, on which it is probably partially based. Again, we find the mysterious blinding of a Greek warrior, or hoplite (here named Polyzelus rather than Epizelus), at Marathon after he saw a ‘supernatural vision’ (ὑπεράνθρωπον φαντασίαν).^27^ The most important (now-lost) source for the story in the ancient world was on the Stoa Poikile, or ‘Painted Stoa’, in Athens.^28^ Here, Aelian (*On Animals* 7.38) records that Epizelus was portrayed in combat with the Persians, alongside a handful of conspicuous fighters at Marathon, on a painting of the battle that was finished in the later 460s and so produced before Herodotus’ *Histories*.^29^ This monument, according to Aeschines (3.186), was specifically designed to glorify the deeds of the Athenians at Marathon.^30^

The Epizelus story is drawn along the lines of a traditional folk-history tale, with a central hero, a kind of *peripeteia*, or dramatic reversal, and the intervention of the supernatural in human affairs.^31^ The key to understanding the passage is not in the nature of the wound but in the role of the large hoplite *phasma* at the apex of the narrative in 6.117. The figure features as a Greek hoplite, with a classic Greek *aspis* shield, but he does not fight on Epizelus’ side:^32^ he fights Greeks, helping the Persian effort, yet he also clearly chooses not to harm Epizelus, whose blinding, Herodotus observes, was not caused by the great hoplite.^33^ The ambiguous positioning of the hoplite has posed a difficulty for scholars who have read the story as a record of psychogenic trauma: they must (mis)characterise the hoplite as a malevolent figure, often as a Persian, who represents an overwhelming trauma-causing danger.^34^

Attempts to reconcile the figure as Greek-but-enemy have been unconvincing. For instance, Zoja argues that the figure is ‘surely an enemy of the Greeks’, who represents elements of ‘collective psychology’: the Greek fear of espionage from within their ranks in a war in which Greeks fought alongside the Persians against other Greeks.^35^ But Greeks commonly fought other Greeks throughout Greek history, and any sense of panhellenic identity in 490 BC was fragile at best: the construction of Greekness in opposition to the barbarian mostly took place in the Early Classical Period (from 480 BC), in reaction to the Persian Wars.^36^ By far the strongest statement of panhellenic identity in opposition to the barbarian is a ‘rhetorical construct’ of Herodotus (8.144) predicated on its insecurity.^37^ It is voiced by an Athenian spokesman in the winter of 480/79 BC in response to the Spartan assumption that the Athenians would defect to the Persians (which they later threaten to do, in Hdt 9.11), and was promptly followed by an attack on the Spartans for their own lack of solidarity with Greeks. Against the rhetorical construction of panhellenic identity, Herodotus presents Greek *poleis* as fundamentally self-interested (as they also are in Thucydides).^38^

There is little in all of this to suggest that Greeks fighting the Persians would have felt an elevated ‘internal threat’ from individual Greek *poleis* (city-states) choosing to ally with the Persians. As such, it would be surprising if the presence of Greeks fighting on the Persian side had any significant collective psychological consequences, as Zoja argues;^39^ and it seems incredibly unlikely that it could have been a driver or mediator for any specific individual psychogenic trauma of Epizelus. Proponents of this view, as with all readers who argue that the hoplite was essentially hostile to Epizelus, cannot reconcile this hostility with several aspects of the narrative and are likely to conclude that Epizelus’ own explanation of his experience in Herodotus makes no apparent sense’ and is ‘quite irrational’.^40^

It is difficult to explain a human dressed like a Greek but fighting on behalf of the Persians, who spared Epizelus but not the other Athenians around him, but there is nothing unusual about gods warring on behalf of each side of a battlefield and selectively killing Greeks.^41^ The reluctance to harm Epizelus only makes sense if the great hoplite was a superhuman force, like a god: the notion that the gods avoided killing good and pious men when they could, even when fighting against their factions, is found as early as in the *Iliad* and is consistent with Herodotus’ own conception of the gods elsewhere.^42^ That Greeks may have believed that Epizelus continued to fight after he had been blinded (which is likely how he was depicted on the Stoa) offered a kind of post-hoc justification: a valorous and tough act that explains why the god spared him.^43^ The description of the hoplite is also markedly god-like: Herodotus described him as a *phasma*, a phantom or apparition, which prohibits interpretation of this figure as a human Greek combatant fighting on the Persian side and indicates a superhuman force.^44^ Other features that mark his divinity include his large stature and his impractically long beard.^45^

While scholars on Greek religion typically do not interpret this passage in Herodotus’ *Histories* as one with deep religious meaning, it has been read as a supernatural epiphany before.^46^ It is generally accepted that ‘[t]he [Epizelus] narrative is built on the general guidelines of an Iliadic battlefield epiphany’.^47^ Indeed, according to Greek tradition there was at least one other superhuman force on the Marathonian battlefield. Pausanias (1.32.5) reports a figure dressed as a farmer, later identified as the hero Echetlaos after the Athenians asked the oracle his identity, Plutarch (*Thes.* 35) claims Theseus led Greeks against the Persians, and in the Stoa Poikile Athenian fighters were depicted ‘commingling with gods’, including Athena and Heracles.^48^ Epizelus’ epiphany is one end of a supernatural bookend for the Marathon narrative, following the first epiphany of Pan at Marathon (Hdt 6.105-6 and Paus. 1.28.4), as Pelling and Hornblower observe.^49^ There are other suggestions of divine presence at Marathon, such as the battle’s location in a sacred area of Herakles (Hdt 6.116.1);^50^ the backdrop of Spartan failure to attend Marathon due to domestic religious obligations (Hdt 6.105);^51^ the theme of unusual changes in light at Marathon that usually marks the appearance of the gods, from the mention of the moon (Hdt 6.106) to the loss of the light in Epizelus’ eyes;^52^ and Epizelus’ abrupt blinding which is the mirror of the usual sudden ‘disappearance’ of gods in epiphanies.^53^

Blindness was connected with transgression against the gods in Greek culture.^54^ The only other case of blindness in Herodotus is King Pheron (2.111): blinded for the impiety of throwing his knife into the Nile, which was an insult to the god.^55^ Beyond Herodotus, seeing the gods is transgressive, and blinding is associated with this.^56^ This aspect is built on the Homeric model: in the *Iliad* (5.121-34), Athene lifts the mist from the eyes of the great soldier Diomedes, so that he could perceive other gods who did not want to be seen, which, alongside other transgressive actions, as Dione remarks, send him mad: a state associated with blindness in Greece.^57^ It is also interesting to observe that soldiers who had been blinded sought divine intervention at the Sanctuary of Asklepius at Epidaurus, where there stood two monumental inscriptions recording the miraculous healing of their temporary battlefield blindness.^58^

Herodotus’ distancing methods in describing the event, writing in *oratio obliqua* and attributing his source as hearsay, is often taken as a signal that Herodotus was not willing to vouch for the accuracy of the story, but this kind of distancing also commonly marks an epiphany and may reflect Herodotus’ broader commitment to the ambiguity and ultimate unknowability of the gods.^59^ The presence of the supernatural in the Epizelus episode is ‘evident’, but it is far from clear exactly what form the supernatural took.^60^ Evelyn Harrison suggested that the apparition was a ‘personification of fear, like *Phobos* and *Deimos*’.^61^ This is not right. The figure is certainly mighty and intimidating, but there is no sense of overpowering fear in the passage: Epizelus is in awe of the god, he is not afraid of him.^62^ Herodotus describes *thoma*, wonder, rather than *phobos*, fear, or *deimos*, panic.^63^ As Hartog has argued, *thoma* gives a sense of ‘otherness’ and inexplicability; a sense of wonder entirely appropriate to this peculiar passage with its mysterious figure and unexplained spontaneous blinding.^64^ A common interpretation of the figure is of Pan, but on this Herodotus’ narrative is ambiguous.^65^ Pan’s offer of help to the Athenians at Marathon in 6.105, with 6.117 as the other bookend of the narrative, might at first glance support this reading. But it does not fit well with other parts of Herodotus’ text, as Pelling and Hornblower have argued: in particular, Pan promised the Athenians help, but, they argue, the hoplite is ‘evidently fighting on the Persian side’.^66^

Hornblower and Pelling’s gloss does not capture the ambiguous role of the figure as we have seen, but it is certainly not true that the figure is helping the Athenians (though they later sought to identify and honour him).^67^ Otherwise, Herodotus’ description does not give much to go on, except for the hoplite’s long beard, which might also indicate an identification of Pan but is really compatible with most divine presences.^68^ Overall, the only thing that seems clear about the figure is that it is probably a god, and we should read the dominant and meaningful aspect of this passage as concerning the epiphany of a god, but Herodotus left the identity, motives, and role of the god conspicuously unexplained.

## Conclusion

The Epizelus episode has been ‘misunderstood’^69^ for several related reasons. Scholars have been concerned with whether the story is ‘true’, ‘factual’, or diagnostic, above how and why it was meaningful to Herodotus, the Athenians, and the Greeks and by extension why it continues to be meaningful to readers today.^70^ The debate has focussed on whether it is acceptable for history to have therapeutic value or for contemporary traumatised veterans to see precursors in the ancient world. These stories of heroic sacrifice set to cosmological and theological themes have resounded with trauma victims since at least the First World War. Exploration of these kinds of Greek stories continues to have real therapeutic value, as explored, for instance, in the dream-healing pilgrimages of Jungian psychotherapist Ed Tick for Vietnam veterans who found meaning in the narratives of heroic Greek mythological archetypal warriors sacrificing at war.^71^

Scholars like Bakogianni and Rees are no doubt correct to warn that we must not confuse the effectiveness of the use of historical culture and literature in treatment for adverse psychological reactions to combat or conditions such as PTSD in the modern world as evidence for these conditions in the ancient world.^72^ Viewing the Epizelus episode as a record of an individual with PTSD or some other specific condition arising from an adverse psychological reaction to combat is engaging in Whig history, and it is presentist, and so it misses the authentic, historical reasons that the story was significant to the Greeks, and the real value of the source material. But it cannot be right to treat the ‘use of ancient history to discuss therapeutic ideas’ in the Epizelus episode as ‘dangerous’.^73^ It is not a choice between historicity or placing the ‘real value’ of these texts in therapeutics and provided the historical element is properly and independently treated, using it to find insight into trauma and recovery is not presentist. That stories like Epizelus’ resound with modern trauma victims should be celebrated: it is a meaningful healing story. Equally, contextualising Epizelus’ vision as a story of divine epiphany does not mean denying that it is also significant for discussions of battlefield trauma.^74^ Recontextualising it in its religious setting will hopefully help scholars in health history to place the episode with other material and scholarship on religious worldview and trauma.

## Footnotes

1 Hdt 6.117, De Sélincourt’s translation, heavily adapted.

2 Dean A. Worcester, ‘Shell-Shock in the Battle of Marathon’, *Science*, 1919, 50, 230, at 230.

3 P. S. Ellis, ‘The Origins of the War Neuroses’, *Journal of the Royal Naval Medical Service*, 1984, 70, 168–77, at 168; S. Regel and S. Joseph, *Post-traumatic Stress* (Oxford: OUP, 2010), 4.

4 J. Hudson, ‘The First Case of Battle Hysteria?’, *The British Journal of Psychiatry*, 1990, 157, 150; Heen King, ‘Recovering Hysteria from History: Herodotus and the First Case of “Shell-Shock”’, in Peter W. Halligan, Christopher Bass and John C. Marshall, eds, *Contemporary Approaches to the Study of Hysteria* (Oxford University Press, 2001), 36–48, at 36–48; E. R. Parson, ‘Post-Traumatic Stress Disorder: Its Biopsychobehavioral Aspects and Management’, in B. Wolman and G. Stricker, eds, *Anxiety and Related Disorders: A Handbook* (New York: John Wiley, 1994), 226–385; Lionel Scott, *Historical Commentary on Herodotus, Book 6*, (Leiden: Brill, 2005), 395–6; Lawrence A. Tritle, ‘Warfare in Herodotus’, in C. Dewald and J. Marincola, eds, *The Cambridge Companion to Herodotus* (Cambridge: Cambridge University Press, 2006), 209–23, at 214–5.

5 Victor Davis Hanson, *The Western Way of War: Infantry Battle in Classical Greece* (London New York: Hodder & Stoughton, 1989), 192.

6 M. A. Crocq and L. Crocq, ‘From Shell Shock and War Neurosis to Posttraumatic Stress Disorder: A History of Psychotraumatology’, *Dialogues in Clinical Neuroscience*, 2000, 2, 47–55, at 47.

7 Hans van Wees, *Greek Warfare: Myths and Realities* (London: Duckworth, 2004), 151; Peter Krentz, *The Battle of Marathon* (New Haven: Yale University Press, 2010), 157; Lawrence A. Tritle, *From Melos to My Lai: War and Survival* (London: Routledge, 2000), 64; Lawrence A. Tritle, ‘“Ravished Minds” in the Ancient World’, in P. Meineck and D. Konstan, eds, *Combat Trauma and the Ancient Greeks (The New Antiquity)* (New York: Palgrave Macmillan US, 2014), 87–103, at 88. Conversion disorder: A. Greaves, ‘Post-Traumatic Stress Disorder (PTSD) in Ancient Greece: A Methodological Review’ (2013), 89–100, at 93; S. Pridmore, ‘Herodotus on Conversion Disorder’, *Australian & New Zealand Journal of Psychiatry*, 2014, 48, 1170–74; F. S. Naiden, ‘Religion and Warfare’, in Waldemar Heckel *et al*., eds, *A Companion to Greek Warfare* (London: Blackwell, 2021), 312–28, at 323. See also R. J. Brown *et al*., ‘Should Conversion Disorder Be Reclassified as a Dissociative Disorder in DSM V?’, *Psychosomatics*, 2007, 48, 369–78 on similar types of ‘conversion reactions’ in modern patients; also covered by Yulia Ustinova and Etzel Cardeña, ‘Combat Stress Disorders and Their Treatment in Ancient Greece’, *Psychological Trauma: Theory, Research, Practice, and Policy*, 2014, 6, 739–48, at 742.

8 Philippe J. Birmes and Eric Bui, ‘PTSD in History: From Uruk to Baghdad’, in Colin R. Martin, Victor R. Preedy, and Vinood B. Patel, eds, *Comprehensive Guide to Post-Traumatic Stress Disorders* (Cham: Springer International Publishing, 2016), 3–19, at 4; W. K. Abdul-Hamid and J. H. Hughes, ‘Nothing New under the Sun: Post-traumatic Stress Disorders in the Ancient World’, *Early Science and Medicine*, 2014, 19, 549–57, at 551. On the causes of PTSD, see William P. Nash, ‘The Stressors of War’, in Charles R. Figley and William P. Nash, eds, *Combat Stress Injury: Theory, Research, and Management* (London: Routledge, 2007), 11–32.

9 Medical interest: e.g. Hudson, ‘The First case of Battle Hysteria?’ Luigi Zoja, *Growth and Guilt: Psychology and the Limits of Development* (London: Routledge, 1995); Crocq and Crocq, ‘From Shell Shock and War Neurosis to Posttraumatic Stress Disorder: A History of Psychotraumatology’; Regel and Joseph, *Post-traumatic Stress*, 4–5; Ustinova and Cardeña, ‘Combat Stress Disorders and Their Treatment in Ancient Greece’ and Abdul-Hamid and Hughes, ‘Nothing New under the Sun: Post-traumatic Stress Disorders in the Ancient World’. Psychiatrists have offered confident clinical diagnoses of Epizelus, like that of G. S. Philippopoulos, ‘An Early Case of Battle Hysteria’, *British Journal of Psychiatry*, 1981, 139, 82–83, at 83: ‘the description is so clear and convincing that the clinical diagnosis of a battle conversion can hardly be argued’.

10 A. Griffiths, ‘Was Kleomenes Mad?’, in Anton Powell, ed, *Classical Sparta: The Techniques Behind Her Success* (London: Croom Helm, 1988), 51–78, at 72.

11 Lawrence Tritle, ‘Battle Trauma in Ancient Greece’, in Waldemar Heckel *et al*., eds, *A Companion to Greek Warfare* (London: Blackwell, 2021), 301–11, at 302–3 observes, perhaps optimistically, that there is now broad consensus in the medical community, and ‘growing acceptance’, that ancient Greeks experiences battlefield trauma.

12 Jason Crowley, ‘Beyond the Universal Soldier: Combat Trauma in Classical Antiquity’, in Peter Meineck and David Konstan, eds, *Combat Trauma and the Ancient Greeks* (New York: Palgrave Macmillan US, 2014), 105–30, at 105–15; Greaves, ‘Post-Traumatic Stress Disorder (PTSD) in Ancient Greece: A Methodological Review’, 90–1.

13 On the universality of combat trauma, see Greaves, ‘Post-Traumatic Stress Disorder (PTSD) in Ancient Greece: A Methodological Review’, arguing evidence of this ubiquitous phenomenon filters through Greek culture; D. Konstan, ‘Introduction Combat Trauma: The Missing Diagnosis in Ancient Greece?’, in P. Meineck and D. Konstan, eds, *Combat Trauma and the Ancient Greeks* (New York: Palgrave Macmillan, 2014), 1–14, arguing that the failure to individualise and pathologise combat trauma by the Greeks reflects its ubiquity and association with valour; and Tritle, ‘“Ravished Minds” in the Ancient World’, esp. 96–8, who describes the idea that battle trauma is a contemporary phenomenon as ‘preposterous’, ‘tragic’, ‘folly’ (87) as it is grounded in underlying human physiology triggered by ‘simple combat stress’; B. Steinbock, ‘“Sufferings Too Great for Tears”: The Destruction of the Athenian Expeditionary Corps in Sicily’, in Melanie Jonasch, ed, *The Fight for Greek Sicily: Society, Politics, and Landscape* (Philadelphia: Oxbow Books, 2020), 73–98, at esp. 76–81, claims scholars who deny the universality of combat trauma are contradicted by the evidence and stereotype ancient warfare.

14 Konstan, ‘Introduction Combat Trauma: The Missing Diagnosis in Ancient Greece?’ on the societal valourising of traumatised men; Tritle, ‘“Ravished Minds” in the Ancient World’, 88, on ‘culturally available’ terms. Owen Rees, ‘We Need to Talk About Epizelus: “PTSD” and the Ancient World’, *Medical Humanities*, 2020, 46, 46, at 51 questions whether Epizelus was heroisd rather than traumatised, but the opposition is artificial.

15 Greaves, ‘Post-Traumatic Stress Disorder (PTSD) in Ancient Greece: A Methodological Review’; Tritle, ‘“Ravished Minds” in the Ancient World’, 88–9.

16 Katie Batza, ‘LGBTQ Experiences and Health’, in Katherine Crawford-Lackey and Megan E. Springate, eds, *Communities and Place: A Thematic Approach to the Histories of LGBTQ Communities in the United States* (Berghahn Books, 2020), 150–77; Virginia Berridge, ‘Historical Research’, in Pauline Allen *et al*., eds, *Studying the Organisation and Delivery of Health Services* (London: Routledge, 2001), 140–53, at 145; P. R. Thompson and R. Perks, *An Introduction to the Use of Oral History in the History of Medicine* (London: The British Library National Sound Archive, 1993).

17 G. Scally and J. Womack, ‘The Importance of the Past in Public Health’, *Journal of Epidemiology and Community Health*, 2004, 58, 751–5, at 751. Sanjoy Bhattacharya, Alexander Medcalf and Aliko Ahmed, ‘Humanities, Criticality and Transparency: Global Health Histories and the Foundations of Inter-Sectoral Partnerships for the Democratisation of Knowledge’, *Humanities and Social Sciences Communications*, 2020, 7, 1–11, at 2.

18 James Nicholls and James Kneale, ‘Alcohol Problems and Policies: Historical and Contemporary Perspectives’, *Drugs: Education, Prevention and Policy*, 2015, 22, 93–95, at 95.

19 Berridge, ‘Historical research’, 151; Virginia Berridge, ‘Making Public Health History Usable: The Launch of a New Series in JECH’, *Journal of Epidemiology and Community Health*, 2007, 61, 90–1, at 90; Virginia Berridge, ‘Why Policy Needs History (and Historians)’, *Health Econ Policy Law*, 2018, 13, 369–81, at 379; S. Galea, ‘History as a Determinant of Health’, *Dean’s Notes, Boston University School of Public Health*, 2017, 1–5; Richard F. Hirsh, ‘Historians of Technology in the Real World: Reflections on the Pursuit of Policy-Oriented History’, *Technology and Culture*, 2011, 52, 6–20, at 20; Scally and Womack, ‘The Importance of the Past in Public Health’, 751.

20 Bhattacharya, Medcalf and Ahmed, ‘Humanities, Criticality and Transparency: Global Health Histories and the Foundations of Inter-Sectoral Partnerships for the Democratisation of Knowledge’, 1; Galea, ‘History as a Determinant of Health’; D. S. Jones *et al*., ‘Making the Case for History in Medical Education’, *Journal of the History of Medicine and Allied Sciences*, 2015, 70, 623–52.

21 Historiography: Virginia Berridge and John Stewart, ‘History: A Social Science Neglected by Other Social Sciences (and Why It Should Not Be)’, *Contemporary Social Science*, 2012, 7, 39–54, at 48; Brigid Lusk, ‘Historical Methodology for Nursing Research’, *Image: The Journal of Nursing Scholarship*, 1997, 29, 355–60, at 355. New aspects: Berridge, ‘Making Public Health History Usable: The Launch of a New Series in JECH’, 90; Berridge, ‘Why Policy Needs History (and Historians)’, 379–80; Hirsh, ‘Historians of Technology in the Real World: Reflections on the Pursuit of Policy-Oriented History’, 20; Catherine Pope and Nick Mays, ‘Qualitative Research: Reaching the Parts Other Methods Cannot Reach: An Introduction to Qualitative Methods in Health and Health Services Research’, *BMJ*, 1995, 311, 42–45, at 4.

22 Critiquing and processing quantitative data: Berridge, ‘Historical Research’, 144; Pope and Mays, ‘Qualitative Research: Reaching the Parts Other Methods Cannot Reach: An Introduction to Qualitative Methods in Health and Health Services Research’, 5.

23 Virginia Berridge and P. Strong, ‘AIDS and the Relevance of History’, *Social History of Medicine*, 1991, 4, 129–38, at 138; Lusk, ‘Historical Methodology for Nursing Research’, 355; Bas J. P. van Bavel *et al*., ‘Climate and Society in Long-Term Perspective: Opportunities and Pitfalls in the Use of Historical Datasets’, *WIREs Climate Change*, 2019, 10, 1–18, at 2.

24 On historians as ‘marginal natives’ in health: M. Hammersley and P. Atkinson, *Ethnography: Principles in Practice* (London: Routledge, 2019); Virginia Berridge, ‘History in Public Health: Who Needs It?’, *Lancet*, 2000, 356, 1923–5, at 1925; Virginia Berridge, ‘History in Public Health: a New Development for History?’, *Hygiea Internationalis: An Interdisciplinary Journal for the History of Public Health*, 2000, 1, 23–35, at 34; Virginia Berridge, ‘“Hidden from History”?: Oral History and the History of Health Policy’, *Oral History*, 2010, 38, 91–100, at 96; Alan Bleakley, ‘Introduction. The Medical Humanities: A Mixed Weather Front on a Global Scale’, in Alan Bleakley, ed, *Routledge Handbook of the Medical Humanities* (London: Routledge, 2019), 1–28, at 21–4; Scally and Womack, ‘The Importance of the Past in Public Health’, 754.

25 Charles W. Fornara, ‘Evidence for the Date of Herodotus’ Publication’, *The Journal of Hellenic Studies*, 1971, 91, 25–34 argues for as late as 414BC; Justus Cobet, ‘Wann Wurde Herodots Darstellung der Perserkriege Publiziert?’, *Hermes*, 1977, 105, 2–27 disagrees with Fornara; Charles W. Fornara, ‘Herodotus’ Knowledge of the Archidamian War’, *Hermes*, 1981, 109, 149–56 in reply to Cobet; David Sansone, ‘The Date of Herodotus’ Publication’, *Illinois Classical Studies*, 1985, 10, 1–9 attempting a reconciliation by arguing some of the *Histories* was published before 425 BC, with other parts produced after; E. Irwin, ‘“The Hybris of Theseus” and the Date of the Histories’, in K. Ruffing and B. Dunsch, eds, *Source References in Herodotus – Herodotus’ Sources: Conference in memoriam Detlev Fehlin* (Wiesbaden: Harrasowitz Verlag, 2013), 7–93; and Elizabeth Irwin, ‘The End of the Histories and the End of the Atheno-Peloponnesian Wars’, in *Interpreting Herodotus* (Oxford: Oxford University Press, 2018), 279–334 expand Fornara’s argument, arguing for a publication date of 403BC.

26 Lawrence A. Tritle, ‘Soldiers’ Home’, in Lee L. Brice, ed, *New Approaches to Greek and Roman Warfare* (Wiley, 2020), 53–64, at 57.

27 [Plut.] *Parallel Minora*, Datis and Hasdrubal 1/305b-c.

28 A full reconstruction of the painting is impossible, but a general one is attempted in Mark Stansbury-O’Donnell, *Pictorial Narrative in Ancient Greek Art* (Cambridge: Cambridge University Press, 1999), 142–3, building on Evelyn B. Harrison, ‘Preparations for Marathon, the Niobid Painter and Herodotus’, *The Art Bulletin*, 1972, 54, 390–402. A more recent breakdown is Olga Palagia, ‘The Iconography of War’, in Waldemar Heckel *et al*., eds, *A Companion to Greek Warfare* (Blackwell: London, 2021), 369–83, at 370–1.

29 In the stoa sources—Aelian *On Animals* 7.38, Paus. 1.15.3—very few ‘conspicuous’ (as in Paus.) fighters are mentioned: Epizelus (Aelian), Cynegirus (Aelian), Callimachus (Aelian, Paus.), Miltiades (Paus.) and the local hero Echetlaos (Paus.). On the size, date, and authorship of the Stoa Poikile, and its role in a Kimonian program see E. D. Francis, *Image and Idea in Fifth Century Greece: Art and Literature After the Persian Wars*, 1 edn (Florence: Routledge, 1990), 89; Stansbury-O’Donnell, ‘The Painting Program in the Stoa Poikile’, 76–9, 81; Sarah P. Morris, *Daidalos and the origins of Greek Art* (Princeton, NJ: Princeton University Press, 1992), 312–3; E. D. Francis and Michael Vickers, ‘The Oenoe Painting in the Stoa Poikile, and Herodotus’ Account of Marathon’, *The Annual of the British School at Athens*, 1985, 80, 99–113, at 100; Martin Robertson, *A History of Greek Art* (Cambridge: Cambridge University Press, 1975), 243–4, 324; and David Castriota, *Myth, Ethos, and Actuality: Official Art in Fifth-Century B.C. Athens* (Madison: University of Wisconsin Press, 1992), 76–82.

30 Aeschines’ speech is in the context of a comparison between the glorified ancient city and its current dilapidated state, to support a renewal project: see Fiona Hobden, ‘Imagining Past and Present: A Rhetorical Strategy in Aeschines 3, “Against Ctesiphon”’, *The Classical Quarterly*, 2007, 57, 490–501. *Cf.* Demosthenes’ 59.94 reference to the painting as a ‘memorial of their valour’. On the significance of the painting: Robertson, *A Shorter History of Greek Art*, 106; Francis, *Image and Idea in Fifth Century Greece: Art and Literature After the Persian Wars*, 85; Francis and Vickers, ‘The Oenoe Painting in the Stoa Poikile, and Herodotus’ Account of Marathon’, 99.

31 The blinding is reminiscent of Paul’s conversion: *Acts* 9:3-19, see also *1Cor* 15:3-8, *Gal.* 1:11-6, as W. W. How and J. Wells, *A Commentary on Herodotus with Introduction and Appendixes. Books V-IX, Vol. 2* (Oxford: Clarendon Press, 1912), on 6.117; on *topoi* see O. Murray, ‘Herodotus and Oral History’, in Nino Luraghi, ed, *The Historian’s Craft in the Age of Herodotus* (United Kingdom: Oxford University Press, 2007), 16–44, at 31–2; and Murray, ‘Herodotus and Oral History Reconsidered’, 314–25, esp. on cultural value vs historicity of oral histories. Donald Lateiner, ‘Deceptions and Delusions in Herodotus’, *Classical Antiquity*, 1990, 9, 230–46, at 235 observes that the story is a supernatural appearance reported alongside others, ‘as if they were ordinary’.

32 He is not a ‘generic soldier figure’ as Arthur Keaveney and Adam Bartley, ‘The Case of Epizelos (Herodotus 6.117) Revisited’, *Giornale Italiano di Filologia*, 2014, 66, 9–26, at 21.

33 Scott, *Historical Commentary on Herodotus, Book 6*, 396.

34 Ustinova and Cardeña, ‘Combat Stress Disorders and Their Treatment in Ancient Greece’, 742–3 for instance says that Epizelus ‘perceived the giant phantom as hostile to the Greeks’. E.g. Greaves, ‘Post-Traumatic Stress Disorder (PTSD) in Ancient Greece: A Methodological Review’, 93 assumes the figure was a Persian. He is an ‘enemy soldier’ in Tritle, ‘Warfare in Herodotus’, 214.

35 Zoja, *Growth and Guilt: Psychology and the Limits of Development*, 76.

36 E. Hall, *Inventing the Barbarian: Greek Self-definition Through Tragedy* (Oxford: Clarendon Press, 1989).

37 Erich S. Gruen, ‘Herodotus and Greekness’, in *Ethnicity in the Ancient World – Did It Matter?* (Berlin, Boston: De Gruyter, 2020), 42–55.

38 Philip A. Stadter, ‘Thucydides as “Reader” of Herodotus’, in Edith Foster and Donald Lateiner, eds, *Thucydides and Herodotus* (Oxford: Oxford University Press, 2012), 39–66, at 52.

39 Zoja, *Growth and Guilt: Psychology and the Limits of Development*, 76.

40 No sense: *ibid*., 75.. Irrational: N-A. Smaro, ‘ἱστορέειν and θωμάζειν: Scientific Terms and Signs of Unity in Herodotus’ Histories’, in Ewen Bowie, ed, *Herodotus: Narrator, Scientist, Historian* (Germany: Walter de Gruyter GmbH, 2018), 223–42, at 229. As C. B. R. Pelling, Simon Hornblower and Herodotus, *Histories. Herodotus, Book VI* (Cambridge: Cambridge University Press, 2017), 260 have argued, the figure is fighting on behalf of the Persian side in a sense, but it is reductive to call him an enemy.

41 Helping friends was a part of Greek morality: see K. J. Dover, *Greek Popular Morality in the Time of Plato and Aristotle* (Hackett, 1994), 180–4; and more recently Mary Whitlock-Blundell, *Helping Friends and Harming Enemies: A Study in Sophocles and Greek Ethics*, (Cambridge: Cambridge University Press, 1989). Crowley, ‘Beyond the Universal Soldier: Combat Trauma in Classical Antiquity’, 113 argues that ‘there is no hint of pacifism in Greek religion’ so violent behaviour of gods on the battlefield was expected.

42 E.g. Eur. *Hipp.* 1340–1: ‘The gods get no pleasure from the death of good men, it is the wicked we destroy, children, house, and all.’ Likewise even when fated for divine retribution in Hdt 1.34.1 as the fifth generation descendant of the tyrant Gyges, Hdt 1.13.2; Apollo saves Croesus from death in 1.87.2 after having delayed his fall, as confirmed by the Delphic Oracle in 1.91.2-3. Cf. Achilles’ comment to Lycaon in the *Iliad* 21.106-113, that good men are not always saved from death by the gods.

43 The figures on the stoa were unnamed, so to be identifiable as Epizelus he must have had identifiable features: surely bloodied or disfigured eyes, as suggested by Evelyn B. Harrison, ‘The South Frieze of the Nike Temple and the Marathon Painting in the Painted Stoa’, *American Journal of Archaeology*, 1972, 76, 353–78, at 367–8, and Rees, ‘We need to talk about Epizelus: “PTSD” and the ancient world’, 50; and it is difficult to explain his noteworthiness as an injured man, unless he was continuing to fight. as proposed in the Suda s.v. Hippias, which states Epizelus continued fighting after blinding, identifying enemies by voice: a detail not mentioned by Herodotus, so possibly a tradition drawn from the stoa. Paus. 1.15.3 also describes the depiction on the stoa as two sides pushing into each other but does not mention injured and dying Greeks, only injured Persians and fighting Greeks.

44 Phasma: Rees, ‘We need to talk about Epizelus: “PTSD” and the ancient world’, 49 states that *phasma* does not generally refer to a god. But as in Georgia Petridou, *Divine Epiphany in Greek Literature and Culture*, 1st edn (Oxford: Oxford University Press, 2016), 107–70 *phasma* can refer to a range of superhuman manifestations including gods, e.g. the *dioscuri* are *phasma* in Paus. 4.16.9. *Phasma* can be used to mark superhuman manifestation and marginality, the ‘extreme and unexplained’, as in *ibid*., 66., including those of uncertain identification, as Herodotus uses it in 6.117, with distancing methods discussed later, see n.59. As such it uncommonly coincides with named beings, except those later identified, as in this case: the Suda s.v. Hippias records that the Athenians considered this *phasma* Pan.

45 Large stature as superhuman: e.g. Hdt 8.37-8, two giant hoplites fight off Persians trying to plunder Delphi. Beards marked seniority as with the *presbutes makropogon*, long-bearded mask of Comedy indicating seniority, in Pollux 4.143-5 and Aristogeiton the ‘big-bearded one’, the senior lover, in Thuc. 6.54.2; see Davidson, ‘Revolutions in Human Time: Age-Class in Athens and the Greekness of Greek Revolutions’, 36–7, 44, 52–5. It marks power, virility (as opposed to white haired seniors, in Pl. *Stat.* 268d-74b), and fertility. In Herodotus 1.175 and 8.104, Athena’s priestess grows a large beard to warn of misfortune, indicating divine authority. See more generally Legras, *Néotês: recherches sur les jeunes Grecs dans l’Egypte ptolémaïque et romaine*, 57–9, 85, 89–92, particularly 92, and 103.

46 The episode has a ‘hint of an epiphany’ in C. Pelling, ‘Herodotus’ Marathon’, in Christopher Carey and Michael Edwards, eds, *Marathon - 2,500 Years: Proceedings of the Marathon Conference 2010, Bulletin of the Institute of Classical Studies. Supplement; 124* (London: Institute of Classical Studies, School of Advanced Study, University of London, 2013), 23–34, at 31, 34; and P. J. Rhodes, ‘The Battle of Marathon and Modern Scholarship’, in Christopher Carey and Michael Edwards, eds, *Marathon - 2,500 Years: Proceedings of the Marathon Conference 2010, Bulletin of the Institute of Classical Studies. Supplement; 124* (London: Institute of Classical Studies, School of Advanced Study, University of London, 2013), 3–21, at 19, also have it as an epiphany, as does Gabriel Herman, ‘Greek Epiphanies and the Sensed Presence’, *Historia: Zeitschrift für Alte Geschichte*, 2011, 60, 127–57, at 136–7, 53. Naiden, ‘Religion and Warfare’, 323 categorises it as ‘combat epiphany’, as does Esther Eidinow, ‘Ancient Greek Smellscapes and Divine Fragrances: Anthropomorphizing the Gods in Ancient Greek Culture’, in Esther Eidinow, Armin W. Geertz, and John North, eds, *Cognitive Approaches to Ancient Religious Experience Ancient Religion and Cognition* (Cambridge: Cambridge University Press, 2022), 69–95, at 72. But, it is not analysed for deeper religious content, e.g. in H. S. Versnel, ‘What Did Ancient Man See When He Saw a God? Some Reflections on Greco-Roman Epiphany’, in D. van der Plas, ed, *Effigies Dei: Essays on the History of Religions* (Leiden, The Netherlands: Brill, 1987), 42–55 does not mention the Epizelus episode, while Thomas Harrison, *Divinity and History: The Religion of Herodotus* (Oxford: Clarendon Press, 2000), 84 calls the episode ‘curious’ but does not comment on its religious aspects.

47 Petridou, *Divine Epiphany in Greek Literature and Culture*, 114. J. A. S. Evans, ‘Herodotus and the Battle of Marathon’, *Historia: Zeitschrift für Alte Geschichte*, 1993, 42, 279–307: 287 observed that parts of the Marathon narrative have ‘Homeric overtones’. Similarly, Herodotus’ emphasis of the numbers and bravery of the Persians at Plataea, in Hdt 9.62, calls attention to the bravery of the Greeks in defeating them. On epiphanies see Versnel, ‘What Did Ancient Man See When He Saw a God? Some Reflections on Greco-Roman Epiphany’; Petridou, *Divine Epiphany in Greek Literature and Culture*, Verity J. Platt, *Facing the Gods: Epiphany and Representation in Graeco-Roman Art, Literature and Religion* (Cambridge: Cambridge University Press, 2011); Verity J. Platt, ‘Epiphany’, in Esther Eidinow and Julia Kindt, eds, *The Oxford Handbook of Ancient Greek Religion* (Oxford, United Kingdom: Oxford University Press, 2015), 491–504; and Pelling, Hornblower and Herodotus, *Histories. Herodotus, Book VI*, 260–1. Fritz Graf, ‘Trick or Treat? On Collective Epiphanies in Antiquity’, *Illinois Classical Studies*, 2004, 29, 111–30, at 116;

48 J. F. Lazenby, *The Defence of Greece, 490–479 B.C.* (Warminster, England: Aris & Phillips, 1993), 80 argues (unconvincingly) that the stories of Epizelus and Echetlaos were the same story but rightly locates the passage in the context of religion and superstition; Pausanias reports immediately before this in 1.32.3 that visitors to the Marathon battleground at night could hear sounds of battle. Echetlaos: Robert Garland, *Introducing New Gods: The Politics of Athenian Religion* (London: Duckworth, 1992), 62; Versnel, ‘What Did Ancient Man See When He Saw a God? Some Reflections on Greco-Roman Epiphany’, 44–5. Theseus: Herman, ‘Greek Epiphanies and the Sensed Presence’, 137–8. Hugh Bowden, ‘When Pheidippides Met Pan: Problems with Epiphany’, *Bulletin of the Institute of Classical Studies*, 2023, 65, 103–15. On the gods in the Stoa Poikile: Palagia, ‘The Iconography of War’, 371.

49 Pelling, Hornblower and Herodotus, *Histories. Herodotus, Book VI*, 260. See also K. W. Arafat, ‘Marathon in Art’, *Bulletin of the Institute of Classical Studies. Supplement*, 2013, 79–89, at 87. And now on the series of stories about supernatural events (including Epizelus) in the Persian wars: Bowden, ‘When Pheidippides Met Pan: Problems with Epiphany’.

50 Pelling, ‘Herodotus’ Marathon’, 31.

51 Louis Rawlings, *The Ancient Greeks at War* (Manchester: Manchester University Press, 2007), 189.

52 Light games: Ariadne Gartziou-Tatti, ‘Gods, Heroes, and the Battle of Marathon’, *Bulletin of the Institute of Classical Studies. Supplement*, 2013, 91–110, at 95; Jorge Bravo, ‘Heroic Epiphanies: Narrative, Visual, and Cultic Contexts’, *Illinois Classical Studies*, 2004, 29, 63–84, at 66–7. Examples include the story of the divine appearance of Pan to Philippides on the road to Sparta before Marathon, just before the Epizelus passage, in Hdt 6.105-6.

53 Blinding as epiphany: Eidinow, ‘Ancient Greek Smellscapes and Divine Fragrances: Anthropomorphizing the Gods in Ancient Greek Culture’, 72.

54 J. C. Ford, *Atheism at the Agora: A History of Unbelief in Ancient Greek Polytheism*, (London: Taylor & Francis, 2023).

55 Warren R. Dawson and F. D. Harvey, ‘Herodotus as a Medical Writer’, *Bulletin of the Institute of Classical Studies*, 1986, 87–96, at 93–4.

56 Transgressions: 5.850-60. Oedipus ‘look[ed] upon those he should not have seen’: Apollod. *Bib.* 3.5.8; Soph, *OT* 447, 713, 731, 774. Teiresias may be blind but ‘sees the same things as Apollo’, Soph. *OT* 284–5, 1273–4. Justina Gregory, ‘Some Aspects of Seeing in Euripides’ “Bacchae”’, *Greece & Rome*, 1985, 32, 23–31, at 25. Jason is told that he must not look at Hecate: it is implied this transgression would damage him or the rite would fail: Ap. Rhod. *Arg.* 3.1026–62, 1191–1224. Daniel Ogden, *Magic, Witchcraft, and Ghosts in the Greek and Roman Worlds: A Sourcebook* (Cary: Oxford University Press, 2002), 87. Probably the most famous of these kinds of tales is of Lot’s wife; turned into a pillar of salt for turning to watch the destruction of Sodom and Gomorrah: Genesis 19:26. Cf also the story of Orpheus and Eurydice, in Pl. *Symp.* 179d and esp. Paus. 9.30.4-5, ps.-Ap. *Bib.* 1.3.2, Ov. *Met.* 10, Virg. *G.* 4.453.

57 Madness and blindness: e.g. Soph. *OT* 1298–1302. Jean-Pierre Vernant and Pierre Vidal-Naquet, *Tragedy and Myth in Ancient Greece* (Brighton Atlantic Highlands, NJ: Harvester, 1981), 54.

58 Antikrates of Knidos, B 12 (32) in L. R. LiDonnici, ed, *The Epidaurian Miracle Inscriptions: Text, Translation, and Commentary* (Atlanta: Scholars Press, 1995), 109; and Timon, B 20 (40) in *ibid*., 115. Discussed in Tritle, ‘Battle Trauma in Ancient Greece’, 306.

59 Bravo, ‘Heroic Epiphanies: Narrative, Visual, and Cultic Contexts’, 67–8, Guy L. Cooper, ‘Intrusive Oblique Infinitives in Herodotus’, *Transactions of the American Philological Association (1974-)*, 1974, 104, 23–76, at 43; Scott, *Historical Commentary on Herodotus, Book 6*, 395; J. Gould, ‘Herodotus and Religion’, in Simon Hornblower, ed, *Greek Historiography* (Oxford: Clarendon Press, 1994), 91–106, at 96. Unknowability of the gods in Herodotus: Burkert, *Greek Religion: Archaic and Classical*, 131; Burkert, ‘Herodot als Historiker fremder Religionen’, 24–9; Scullion, ‘Herodotus and Greek Religion’, 200.

60 Arafat, ‘Marathon in Art’, 87.

61 Harrison, ‘The South Frieze of the Nike Temple and the Marathon Painting in the Painted Stoa’, 368. Also Ustinova and Cardeña, ‘Combat Stress Disorders and Their Treatment in Ancient Greece’, 743.

62 On the distinction between fear and awe in the history of philosophy see M. Heidegger and W. Kluback, *What Is Philosophy?* (College & University Press, 1956), 80–1 and commentary in Mary-Jane Rubenstein, *Strange Wonder: The Closure of Metaphysics and the Opening of Awe* (Columbia University Press, 2008).

63 As in Hdt 9.120: the prodigy of Protesilaus, where the sentries are afraid of the portent of the cooking fish coming alive, but Artayctes instructs them not to fear because it is a message for him. Divine intervention is not necessarily negative even if frightening: there are obvious biblical comparisons, including the fear and awe of angels at Luke 2:8-10. This sense of awe is also found in the story of the falling tree in Severus 13, on Saint Martin of Tours. Martin wanted to cut down a tree that was sacred to the local populace, so agreed to place himself in its way; but the tree’s trajectory mysteriously changed to avoid Martin, and the locals (who were correspondingly, and surprisingly, nearly crushed) were awed, and convinced to believe in God.

64 François Hartog and Janet Lloyd, *The Mirror of Herodotus: The Representation of the Other in the Writing of History, Translated by Janet Lloyd* (Berkeley: University of California Press, 1988), 231–2, 36–7; Rosaria Vignolo Munson, *Telling Wonders: Ethnographic and Political Discourse in the Work of Herodotus* (Ann Arbor: University of Michigan Press, 2001).

65 E.g. Gartziou-Tatti, ‘Gods, Heroes, and the Battle of Marathon’ observes that the figure has been identified as Pan, as does the Suda s.v. Hippias.

66 Pelling, Hornblower, and Herodotus, *Histories. Herodotus, Book VI*, 260. Similarly, How and Wells, *A Commentary on Herodotus with Introduction and Appendixes. Books V-IX, Vol. 2* on 6.117 remark that it ‘is strange that [Herodotus], unlike Pausanias (i. 15), puts the supernatural aid on the side of the Persians, not of the Greeks.’

67 Paus. 1.32.5; Naiden, ‘Religion and Warfare’, 326.

68 The beard as a marker of power, virility, and fertility suggests the link with Pan. See n.45.

69 Tritle, ‘Warfare in Herodotus’, 214-5.

70 E.g. Bowden, ‘When Pheidippides Met Pan: Problems with Epiphany’.

71 J. Dubisch, ‘Dream Healing for a New Age’, in Steven M. Oberhelman, ed, *Dreams, Healing, and Medicine in Greece: From Antiquity to the Present* (United Kingdom: Routledge, 2016), 317–31, at 321–2; E. Tick, *The Practice of Dream Healing: Bringing Ancient Greek Mysteries Into Modern Medicine* (Quest Books/Theosophical Publishing House, 2001). Critiqued by Rees, ‘We Need to Talk about Epizelus: “PTSD” and the Ancient World’, 47.

72 Anastasia Bakogianni, ‘Shades of Ajax: In Search of the Tragic Hero in Modern War Movies’, in Ricardo Apostol and Anastasia Bakogianni, eds, *Locating Classical Receptions on Screen: Masks, Echoes, Shadows* (Cham: Springer International Publishing, 2018), 147–71, at 157.

73 ‘Dangerous’: Rees, ‘We Need to Talk About Epizelus: “PTSD” and the Ancient World’, 48. Greaves, ‘Post-Traumatic Stress Disorder (PTSD) in Ancient Greece: A Methodological Review’, 97.

74 Presented as such by e.g. Tritle, ‘Soldiers’ Home’, 55.

## Notes

James C. Ford is the Director of the Research Consultancy Stoa Strategy and an Honorary Fellow with the University of Liverpool. In 2023, he published *Atheism at the Agora: A History of Unbelief in Ancient Greek Polytheism*. He has previously worked on the curatorial team at Lancashire County Museums, as a researcher for the Manar Al-Athar project at the University of Oxford, and teaching at the Universities of Manchester and Liverpool.

